# Infectious diseases prevalence, vaccination coverage, and diagnostic challenges in a population of internationally adopted children referred to a Tertiary Care Children's Hospital from 2009 to 2015

**DOI:** 10.1097/MD.0000000000006300

**Published:** 2017-03-24

**Authors:** Sara Sollai, Francesca Ghetti, Leila Bianchi, Maurizio de Martino, Luisa Galli, Elena Chiappini

**Affiliations:** Department of Health Sciences, University of Florence, Meyer Children's University Hospital, Florence, Italy.

**Keywords:** adoption, children, infectious diseases, parasitic infection, tuberculosis

## Abstract

Infectious diseases are common in internationally adopted children (IAC).

With the objective to evaluate infectious diseases prevalence in a large cohort of IAC and to explore possible risk factors for tuberculosis (TB) and parasitic infections, clinical and laboratory data at first screening visit of all IAC (<18 years) consecutively referred to our Center in 2009 to 2015 were collected and analyzed.

In total, 1612 children (median age: 5.40 years; interquartile range: 3.00–7.90) were enrolled, 123/1612 (7.60%) having medical conditions included in the special needs definition. The most frequent cutaneous infections were *Molluscum contagiosum* (42/1612; 2.60%) and *Tinea capitis* (37/1612; 2.30%). Viral hepatitis prevalence was <1% (hepatitis B virus [HBV]: 13 children, 0.80%; hepatitis C virus: 1 child, 0.10%; hepatitis A virus: 6 children, 0.40%). A parasitic infection was diagnosed in 372/1612 (23.10%) children. No risk factors for parasitosis were evidenced. Active TB was diagnosed in 4/1355 (0.3%) children, latent TB in 222/1355 (16.40%). Only 3.7% (51/1355) children had concordant positive tuberculin skin test (TST) and QuantiFERON-TB-Gold In-Tube (QFT-G-IT) results. Risk factors for TST+/QFT-G-IT− results were previous Bacille de Calmette-Guérin vaccination (adjusted odds ratio [aOR]: 2.18; 96% confidence interval [CI]: 1.26–3.79; *P* = 0.006), and age ≥5 years (aOR: 1.49; 95% CI: 1.06–2.11; *P* = 0.02). The proportion of children with nonprotective titers for vaccine-preventable diseases (VPD) ranged from 15.70% (208/1323) for tetanus to 35.10% (469/1337) for HBV.

Infectious diseases were commonly observed in our cohort. The high rate of discordant TST/QFT-G results brings up questions regarding the optimal management of these children, and suggests that, at least in children older than 5 years, only QFT-G-IT results may be reliable. The low proportion of children protected for VPD, confirms importance of a timely screening.

## 1 Introduction

Every year more than 2000 children are internationally adopted in Italy, which represents the country with the second highest rate of adoptions after the United States.^[1]^ The living conditions of these children in their country of origin vary greatly. Most of them reside in orphanages, where they may experience malnutrition, emotional and physical neglect, environmental deprivation, and where they have been susceptible to infectious diseases such as tuberculosis (TB), chronic viral diseases, and parasitic infections.^[2–9]^ Moreover, internationally adopted children (IAC) have often experienced pre- and peri-natal complications, such as exposure to drugs and alcohol during gestation, absence of peri-natal care, low birth weight, and prematurity.^[2–9]^ Although children are declared healthy in their home countries, medical disorders are often missed or diagnosed after adoption: medical preadoption information can be incomplete, wrongly translated and/or discordant.^[2–9]^ Medical care is crucial upon the child's arrival into the country: international adoption medicine is growing for understanding and addressing the specific healthcare needs of these children after their arrival.^[10–12]^ Medical comprehensive evaluations frequently identify unsuspected medical disorders, infections, as well as delayed or incomplete vaccinations: the early identification of infections and lack of protection toward vaccine preventable diseases (VPD) makes treatment of potential transmittable diseases and updating of immunizations possible.^[13–21]^

The majority of the available studies focus on a single issue in selected populations,^[12,22–28]^ whereas few studies are available investigating the prevalence of several pathologies, infectious or not, and immunization status in large cohorts of IAC.^[2,16,29,30]^ Each country has a different geographic profile of IAC, thus medical problems in these children could vary according to their origin. Most of the literature data come from United States studies,^[3–5,9,14,20–22,24,26,28]^ and only few reports have investigated the health status of IAC in Europe.^[2,10,13,15–19,29,31–36]^ Prevalence of medical problems was described in more than 40% of the cases by van Schaik and colleagues in The Netherlands.^[13]^ Other authors found a substantially higher risk of hospitalization or the need for a specialistic consultation in IAC with respect to not-adopted children.^[37]^ Early pubertal development, chronic malnutrition and low weight are frequently reported conditions.^[32,35]^ Hénaff and colleagues reported 55% prevalence of infectious diseases in 133 children adopted in France.^[2]^ Intestinal parasitic and dermatologic infections have been described as the most frequent infectious diseases, with an observed prevalence ranging from 8% to 42.7% and from 22% to 35%, respectively.^[2,10,13,16,33,34,36]^

In the present study, we evaluated the prevalence of infectious diseases in a large cohort of IAC referred to a single center in Italy. Also, we explored possible risk factors for TB and parasitic infections.

## 2 Materials and methods

### 2.1 Study group

All IAC (ages ≤18 years, originating from any foreign country) consecutively referred to the Center for the Internationally Adopted Child of the Infectious Disease Unit (Anna Meyer Children's University Hospital, University of Florence, Italy) in a 7-year period (January 2009 to December 2015) underwent the internal operative protocol for the first screening for IAC and were prospectively enrolled in the study. The only exclusion criterion was being adopted from Italy. Medical records were prospectively collected and entered into an electronic database. Written informed consent to the study was obtained from all the parents of the enrolled children. The study was approved by the Ethics Committee for Human Investigation at the Meyer Children University Hospital.

### 2.2 Screening protocol

All the children underwent an internal standard operative protocol, developed following international recommendations.^[16,38]^ Social-demographic data collected included age on arrival in Italy, age at first evaluation, sex, and country of origin. Family and personal medical history and preadoption immunization records were reviewed when available. Information about possible fetal-alcohol spectrum disorder (FASD), presence of simple or complex malformations, or other clinically relevant pathologies were also registered. Children were considered vaccinated with Bacille de Calmette-Guérin (BCG) whether a clear documentation was available and/or a BCG scar was noted.

All the children were clinically evaluated and pubertal Tanner stage was recorded, as well as the presence of clinical signs of pathology. In case of a cutaneous infection suspicion, a dermatologic evaluation was also performed. At the first evaluation, all the children underwent a venupuncture and laboratory assessment including a complete blood count with differential. Other tests executed included serologic tests for several infectious diseases, and, in particular, hepatitis B virus (HBV), hepatitis C virus (HCV), hepatitis A virus (HAV), human immunodeficiency virus types I and II (HIV 1–2), *Treponema pallidum*, and *Toxocara canis*. Serologic tests to evaluate vaccination coverage for tetanus, diphtheria, measles, mumps, and rubella were also performed. Tuberculin skin test (TST) and QuantiFERON-TB-Gold In-Tube assay (QFT-G-IT) were performed, and a chest radiograph was executed if TST or QFT-G-IT were positive, according to the international guidelines definitions.^[39–41]^ Additionally, 3 stool samples were collected for the search for ova and parasites and for the antigen test for *Giardia lamblia*.

### 2.3 Laboratory tests and TST

All the other laboratory examinations were performed in the same laboratory at the Meyer Children's University Hospital, using standardized techniques and according to manufacturers’ instructions.

Evaluation of HBV surface antigen and HBV surface antibodies, serology for HCV (dosage of HCV antibodies, HCV Ab), HAV (dosage of HAV IgM and IgG), *T pallidum* hemagglutination assay (TPHA), HIV1–2 antibodies and antigen p24 and serologies for measles (measles IgG and IgM), and rubella (rubella IgG and IgM) were performed with chemiluminescent immunoassay (CLIA) technology (LIAISON XL System, DiaSorin, Saluggia [VC], Italy). Serologies for mumps (mumps IgG and IgM), tetanus (tetanus IgG), and diphtheria (diphtheria IgG) were performed with specific enzyme-linked immunosorbent assay (ELISA).

In case of positive serology for HBV or HCV, a polymerase chain reaction (PCR) was performed, with artus HBV PCR Kits CE (QIAGEN, Hilden, Germany).

A diagnosis of giardiasis was performed based on stool ova and parasites examination results on 3 specimens, as previously described.^[23]^ Stool ova and parasites examination was performed with Lugol's staining solution, through direct observation and ×10 and ×40 microscopic enhancement.

A diagnosis of toxocariasis was performed by a serologic test that uses *T canis* excretory-secretory antigen to detect specific IgG antibodies by ELISA. When results were uncertain, Western Blot serology for *T canis* was performed. If concomitant hyper-eosinophilia was detected, specific treatment with albendazole was proposed.

Following the American Academy of Pediatrics guidelines,^[39]^ a positive TST was defined as an induration size ≥10 mm.^[39–41]^ TST was administered by trained nurses working in our Infectious Disease Unit and was performed according to the Mantoux method by injecting intradermally 5 tuberculin units (in 0.1 mL) of purified protein derivative (Statens Serum Institute, Copenhagen, Denmark) into the volar surface of the forearm. The transversed skin induration was recorded (in mm) after 48 to 72 h directly by a pediatrician of the Infectious Disease Unit or by the family physician.

### 2.4 QuantiFERON-TB-Gold In-Tube

The QFT-G-IT assay (Cellestis Inc., Chadstone, Australia) was performed according to the manufacturer's instructions, as previously described. After subtracting the value from the negative control, the result was positive if the antigen-dependent response was ≥0.35 IU, negative if the mitogen-induced response was ≥0.5 IU/mL and the antigen-dependent response was <0.35 IU/mL, and indeterminate if both mitogen-induced and antigen-dependent responses were below cut-off or mitogen-induced response >8 IU/mL. A second test was repeated when an indeterminate result was recorded.

### 2.5 Tuberculosis definition

Study children were classified as not-infected, latent tuberculosis infection (LTBI) cases, or active TB disease cases, following the American Academy Guidelines definition.^[39]^ Asymptomatic children with negative TST and negative QFT-G-IT were defined as uninfected. Confirmed LTBI diagnosis was assigned to any child with positive TST and QFT-G-IT and no clinical or radiographic evidence of active TB.^[39]^ Probable LTBI diagnosis was assigned to any child with positive TST or QFT-G-IT and no clinical or radiographic evidence of active TB.^[39]^ Cases of active TB were defined according to 2 categories: definite TB, children with *Mycobacterium tuberculosis* cultured or detected by microscopy or molecular methods from sputum or gastric aspirate culture; probable TB: absence of microbiological confirmation but presence of all of the following criteria (also taking into account the origin of the child): clinical symptoms and signs of active TB, abnormal radiography and/or computed tomography scan consistent with lung TB, and response to TB therapy.^[39]^

### 2.6 “Children with special needs” definition

As previously reported,^[42–44]^ children were classified as having a “special need” in case of:

(1) diagnosis of psychological or health problems, or a documented condition that may lead to future problems;
(2) age ≥7 years; and
(3) siblings.

### 2.7 Statistical analysis

Data were reported as median and interquartile range (IQR) or absolute numbers and percentages. All continuous variables were not normally distributed thus the nonparametric Mann–Whitney test was used to compare groups. Fisher exact test, Chi-square test or Chi-square for trend tests were used to compare categorical variables, as appropriate. Univariable and multivariable logistic regression analyses were performed to investigate the association between presumed risk factors and intestinal parasitosis, or a positive result of QFT-G-IT, a positive result of TST, and discordant TST+/QFT-G-IT− results. All statistical analyses were carried out using the SPSS (Statistical Package of Social Sciences, Chicago, IL) for Windows software program version 19.0. A *P* value < 0.05 was considered significant.

## 3 Results

### 3.1 Characteristics of the study population

Overall, 1612 children have been included in the study. Median age at first evaluation was 5.3 years (IQR: 3.0–7.9); 961 (59.6%) were males. Most of the children came from a European country (685/1612; 42.5%) and, among them, 436 out of 685 (63.4%) from Russia. Among extra-European countries, IAC most frequently originated from Ethiopia (157/1612; 9.7%), Colombia (122/1612; 7.6%), India (98/1612; 6.1%), and Brazil (87/1612; 5.4%) (Table 1).

**Table 1:**
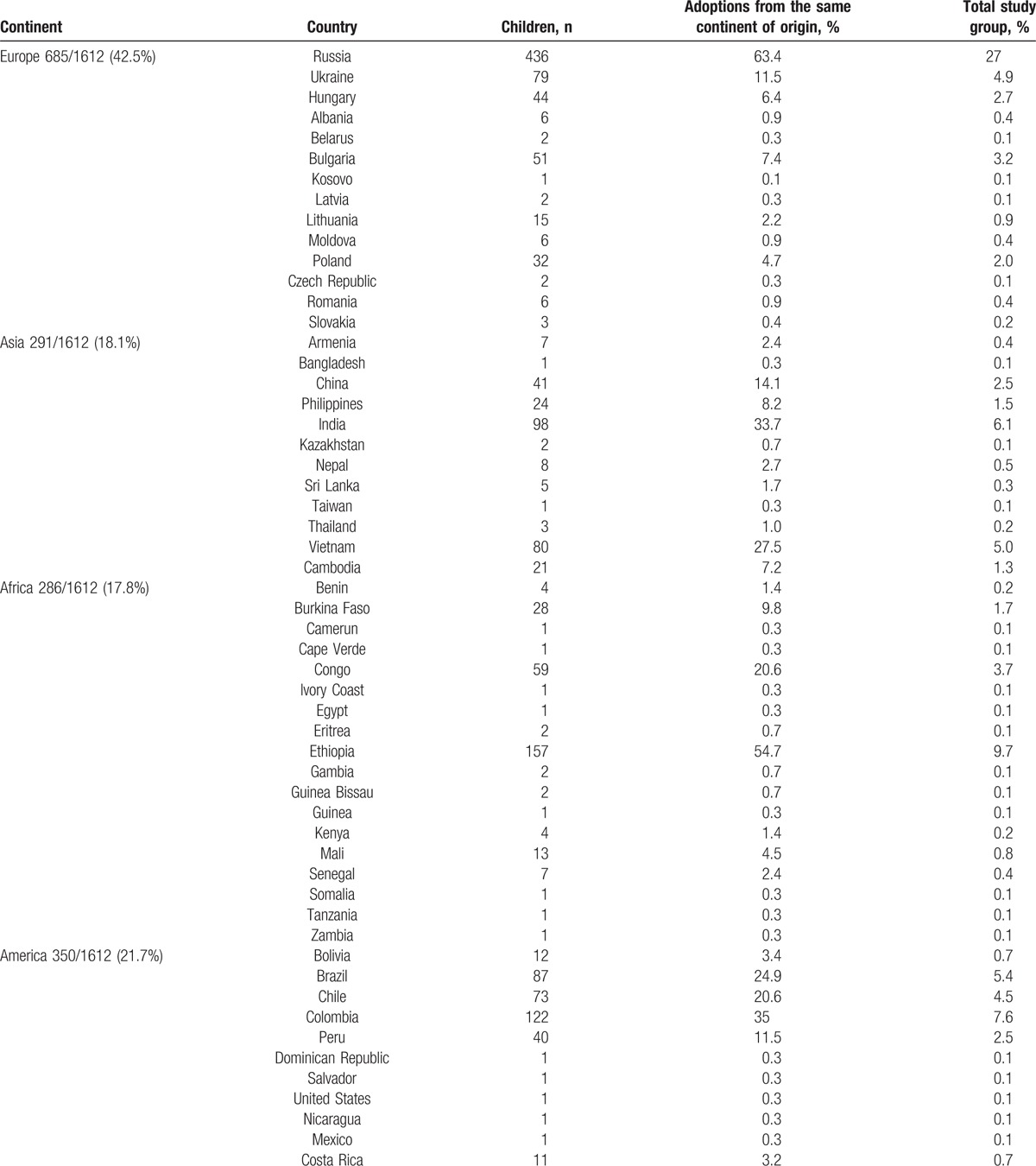
Internationally adopted children classified according to their country of origin.

During the study period we did not observe substantial fluctuations in the distribution of the different origins (χ^2^ for trend 2.565, *P* = 0.11 for African; χ^2^ for trend 0.84, *P* = 0.35 for American; χ^2^ for trend 0.68 *P* = 0.40 for Asian; and χ^2^ for trend 1.26, *P* = 0.26 for European adoptions), with the exception of a significant reduction in the proportion of children coming from Asia in 2011 to 2013 (with homogeneous increase of the adoptions from other continents) with a successive increase in 2014 and 2015. Also, a significant decrease in IAC originating from Africa was observed: the highest proportion was reached in 2013 (54/229; 23.6%) and decreased in 2014 (40/225; 15.7%) and in 2015 (15/198; 7,6%) (χ^2^ for trend 5.04, *P* = 0.02). The characteristics of the study children, classified according to their country of origin, are summarized in Table 2. Children coming from America were older at the time of adoption (median age 7.4 years; IQR: 5.1–9.0) than the children of the other groups (median age 4.7 years; IQR: 2.7–7.2.0; *P* < 0.0001), while African children were the youngest (median age 3.9 years, IQR: 2.0–6.0) (*P* < 0.0001).

**Table 2:**
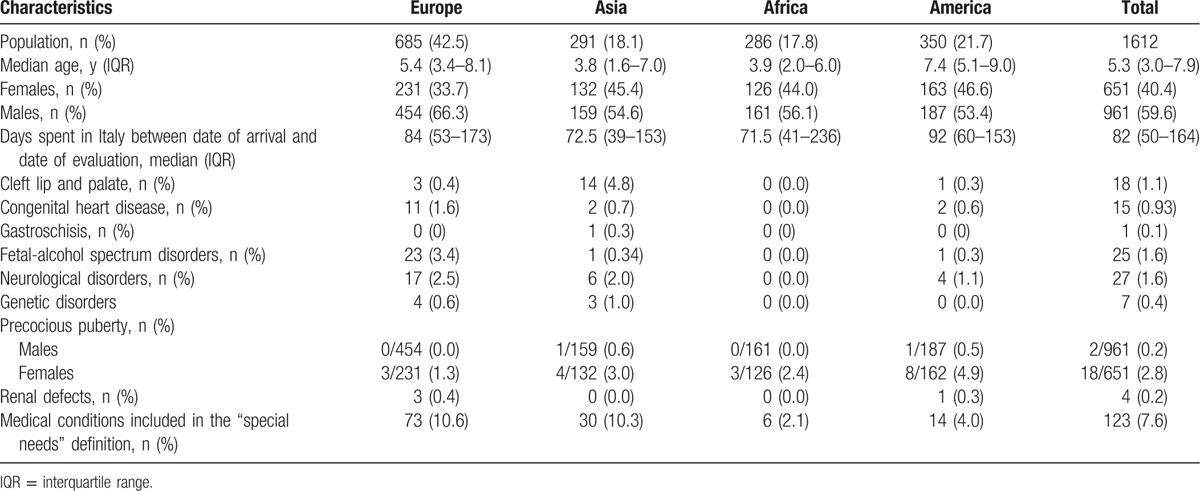
Characteristics of the study children, classified according to country of origin.

Five hundred thirty-eight children out of 1612 (33.4%) were ≥7 years old. Five hundred seventeen children were adopted with siblings (517/1612; 32.1%). In particular, 454/517 (87.8%; 28.2% of the total population) were adopted in couples, while 63/517 (12.2%; 3.91% of the total population) were adopted in groups of 3 siblings. Interestingly, 123/1612 (7.6%) children had medical conditions included in the special needs definition.^[42–44]^ Among them, 56/123 (45.5%) originated from Russia and 21/123 (17.1%) from China. A substantial rate of children with cleft lip and palate was observed among Chinese children (13/18 cleft lip and palate diagnoses; 31.7% of all the adoptions from China), while the majority of children with FASD, major congenital heart disease, genetic syndromes and neurological disorders came from Russia: 10/15 (66.7%) children with congenital heart disease, 22/25 (88%) children with FASD, 4/7 (47.14%) children with genetic disorders, and 13/27 (48.15%) children with neurological disorders (2.3%, 5%, 0.9%, and 3% of all the adoptions from Russia, respectively).

One percent of children (20/1612; 1.2%) displayed precocious puberty, 18/20 (90.0%) being females. The majority of these girls came from South America (8/18; 44.0%).

### 3.2 Infectious diseases prevalence

The most frequent cutaneous infections were *Molluscum contagiosum* (42; 2.6%) and *Tinea capitis* (37; 2.3%). Viral hepatitizes were rarely observed (HBV: 13 children, 0.8%; HCV: 1 child, 0.1%; HAV: 6 children, 0.4%) (Table 3).

**Table 3:**
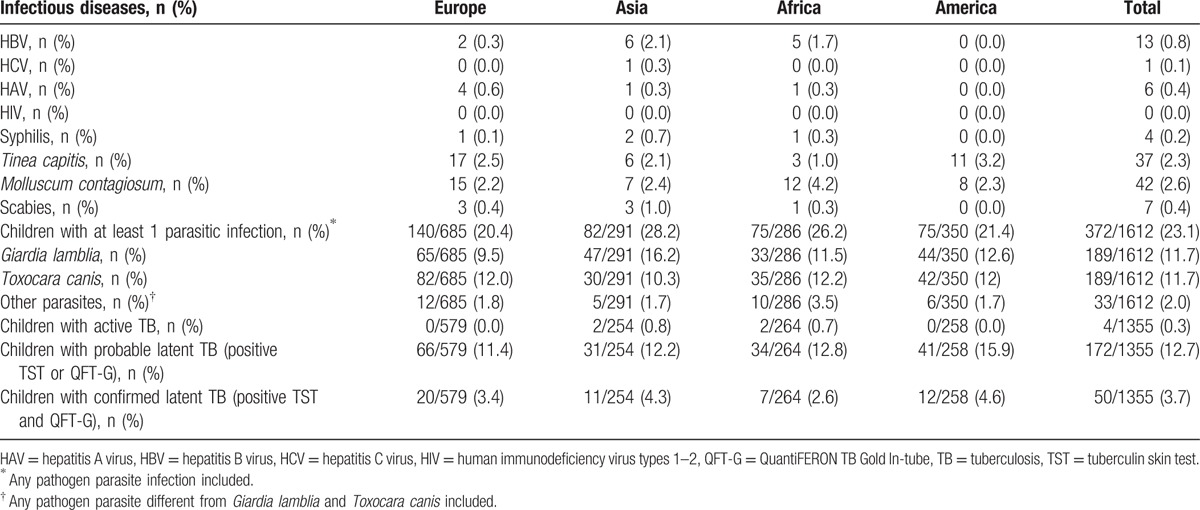
Infectious diseases prevalence in the study group (n = 1612), according to continent of origin.

No child was HIV-infected. Two children (0.1%) (1 South American and 1 European) were known to be born to an HIV-infected mother. Two Asiatic, 1 African, and 1 European child (4/1612; 0.2%) had positive TPHA antibodies for *T pallidum*, with 2 of them also displaying positive VDRL-RPR, requiring treatment with penicillin for probable congenital syphilis (Table 3). Almost one-quarter of children 372/1612 (23.1%) presented with a parasitic infection. Asian (82/291, 28.2%; χ^2^ = 5.20; *P* = 0.02) and African (75/286, 26.2%; χ^2^ = 1.93; *P* = 0.16) children displayed higher rates than the remaining ones. More than 10% of children were positive for *G lamblia* (11.7%) and/or *T canis* (11.7%) infection. Thirty-three children (2.1%) had more than 1 parasitic infection. In particular, 29/1612 (1.8%) children had 2 parasitic infections (3.8% and 2.1% of the Asian and African adoptions, respectively); and 4/1612 had 3 simultaneous parasitic infections (2 European, 1 African, and 1 American children).

Univariable and multivariable analyses were performed to evaluate possible predictors of intestinal parasitosis (Table 4). No significant, independent higher risk for parasitosis was evidenced considering age, sex, the interval between their arrival in Italy and their first clinical evaluation (< or ≥3 months), continent of origin, TB infection diagnosis, and QFT-G-IT (Table 4).

**Table 4:**
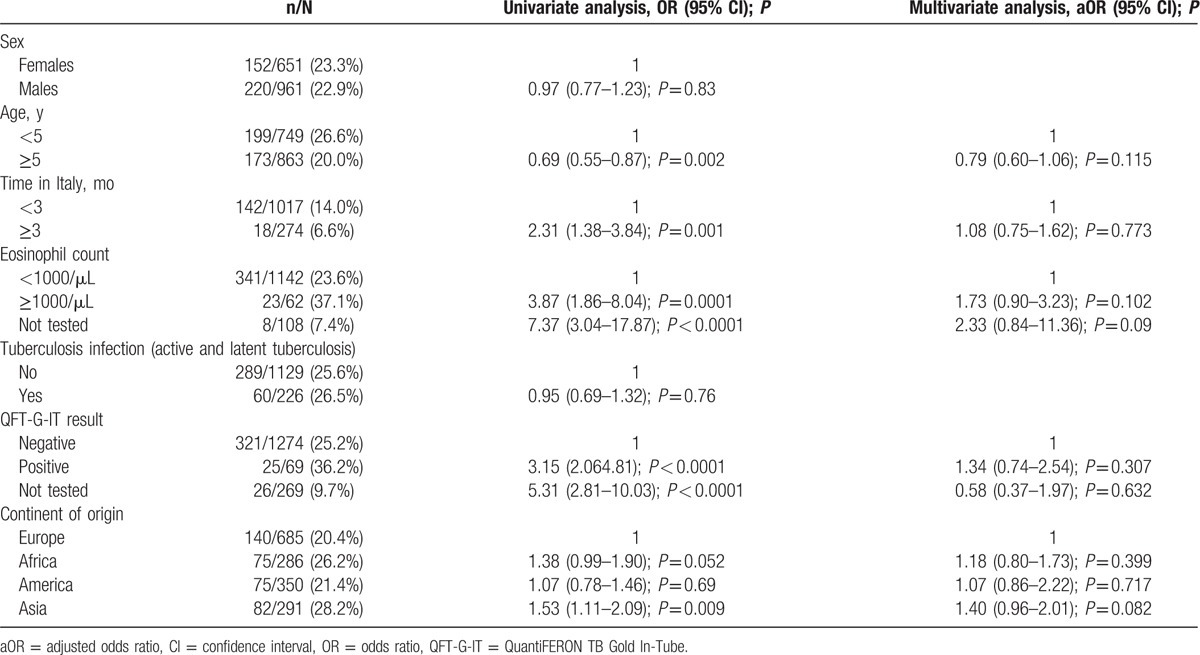
Risk factors for parasitic infection.

### 3.3 Tuberculosis

In the study group, 1355/1612 (84.1%) children were screened for TB. Active TB was diagnosed in 4/1355 (0.3%); 1129 out of 1355 children (83.3%) were classified as not TB infected. Latent TB infection was diagnosed in 222/1355 (16.4%) children, with higher prevalence in the American group (53/258; 20.5%; χ^2^ = 4.02; *P* = 0.04) and European group (86/579; 14.8%; χ^2^ = 1.73; *P* = 0.19). In particular, 50/222 (22.5%) were Confirmed Latent TB, while 172/222 (77.5%) were Probable Latent TB cases.

Of note, only 51/1355 (3.7%) children had concordant positive TST and QFT-G-IT results; while 154/1355 (11.4%) had positive TST and negative QFT-G-IT and 18/1355 (1.3%) had negative TST and positive QFT-G-IT (Table 5). No indeterminate QFT-G-IT result was recorded. Eighty-three out of 154 children (53.9%) with discordant TST+/QFT-G-IT− results came from Eastern Europe. Active TB was diagnosed in 2 children aged <5 and in 2 children aged ≥5. Prevalence of latent TB was similar in the 2 subgroups (12.5% vs 14.8%; *P* = 1.75; χ^2^ = 0.19). We observed a higher frequency (97/732; 13.25% vs 57/623; 9.15%) of positive TST and negative QFT-G-IT discordant results in children aged ≥ 5 versus children aged <5 (*P* = 0.01; χ^2^ = 5.62) and a higher proportion of BCG-vaccinated children in the group ≥5 years versus <5 years (612/732; 83.61% vs 418/623; 67.09%; *P* ≤ 0.0001; χ^2^ = 50.33).

**Table 5:**
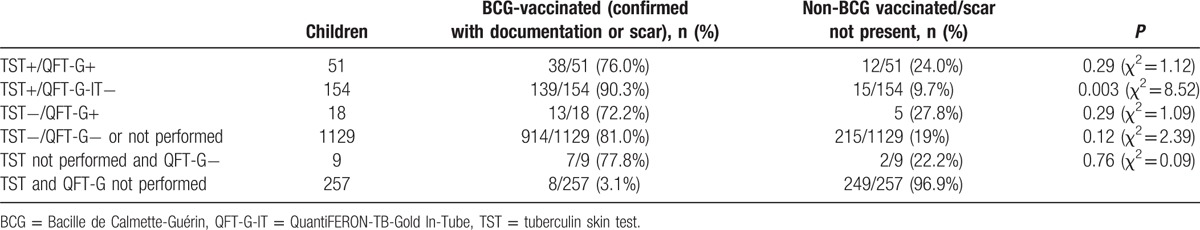
TST and QFT-G-IT test results in BCG vaccinated and nonvaccinated children.

Univariable and multivariable analyses were performed to evaluate possible predictors of discordant results (positive TST and negative QFT-G-IT; Table 6). A significant higher risk for TST+/QFT-G-IT− was related to BCG vaccination (adjusted odds ratio [aOR]: 2.184 [1.26–3.79]; *P* = 0.006) and age ≥5 (aOR: 1.49 [1.06–2.11]; *P* = 0.02) (Table 6).

**Table 6:**
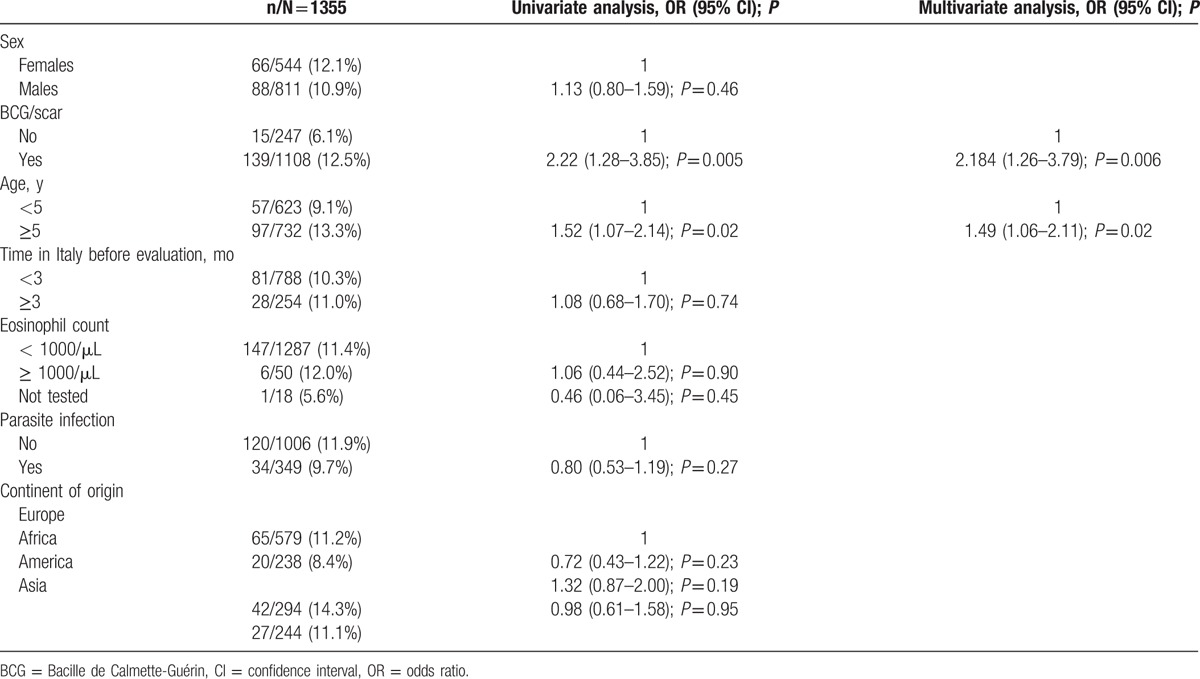
Risk factors for positive tuberculin skin test and negative QuantiFERON-TB-Gold In-Tube results.

At univariable and multivariable logistic regression analyses the only factor significantly associated with a positive TST result was previous vaccination with BCG (aOR 1.56 [95% confidence interval 1.01–2.40]; *P* = 0.04). On the contrary, the only factor associated with a positive QFT result was the lack of a previous BCG vaccination (aOR 0.47 [0.24–0.90]; *P* = 0.023).

### 3.4 Immunization status

Vaccine documentation was available in 743/1612 (46.1%) cases for tetanus and diphtheria, 655/1612 (40.6%) cases for HBV, 668/1612 (41.4%) for measles, and 628/1612 (38.9%) for rubella, and was most frequently available in children originating from Europe. In most children, specific serological tests versus tetanus (1337/1612; 82.1%), diphtheria (170/1612; 10.5%), HBV (1140/1612; 82.9%), rubella (1248/1612; 77.4%), and measles (1243/1612; 77.1%) were performed for evaluating protective or nonprotective antibody titer. A serological test for mumps was performed only in a few cases (25/1612; 1.5%).

**Figure 1:**
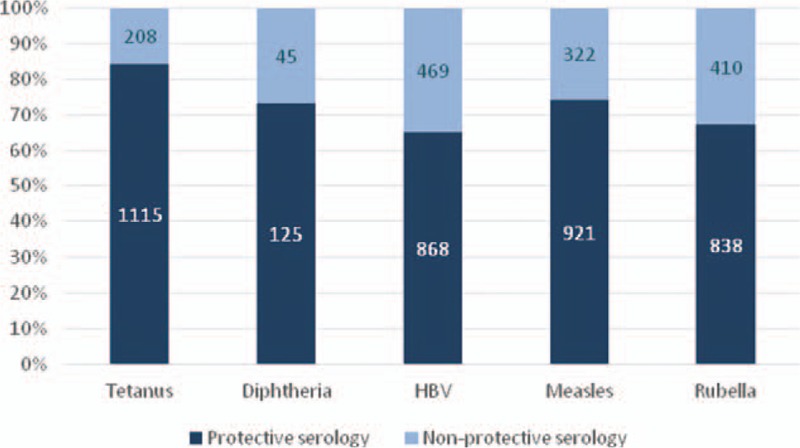
Serologies for the vaccine-preventable infectious diseases. HBV = hepatitis B virus.

In particular, we observed that 15.7% children did not have a protective serology for tetanus (208/1323); 26.4% children were not protected for diphtheria (45/170), and 35.1% for HBV (469/1337). A quarter of the children (25.9%, 322/1243) were not protected for measles, 32.8% were not protected for rubella (410/1248), and 40% for mumps (10/25) (Fig. 1).

Serological tests showed a variable discrepancy between the available documentation and test results. In particular, a nonprotection was documented in 9.0% (67/743) of children considering tetanus, 1.9% (14/743) considering diphtheria, 24.1% (158/655) for HBV, 16.6% (111/668) for measles, and 23.4% (147/628) for rubella.

## 4 Discussion

In the present study we evaluated a population of 1612 IAC (median age 5.4 years, 59.6% males) screened in a tertiary care children's hospital at their arrival in Italy. More than 40% came from a European country and, among them, 63.4% from Russia. Among extra-European countries, IAC most frequently originated from Ethiopia, Colombia, India, and Brazil. No substantial changes in the distribution of the different origins were observed during the study period, except for a significant decrease in IAC originating from Africa in the last 2 years (χ^2^ for trend 5.04, *P* = 0.02). This phenomenon could be related to the decision of the Democratic Republic of Congo to suspend international adoptions on September 2013,^[45]^ a measure that was eliminated in January 2016.^[46]^

The high prevalence (7.6%) of medical conditions included in the special needs definition^[42–44]^ is to be underlined. Almost 50% of these children originated from Russia, 17.1% from China. In particular, the majority of children with FASD (88%), major congenital heart disease (66.7%), genetic syndromes (47.14%), and neurological disorders (48.15%) came from Russia, whereas a substantial rate of children with cleft lip and palate was Chinese (72.22%). One percent of children displayed precocious puberty, 90% being females, and 44% of the cases originating from South America. Infectious diseases were common: 23.1% of the children had at least 1 parasitic infection, mainly by *T canis* and *G lamblia* (more than 10% each), while other parasites were involved in 2% of the cases. Also, cases of multiple (2 or 3) simultaneous parasitosis were recorded (33/1612, 2.0%).

Viral hepatitizes were rarely observed (<1%). No child was HIV-infected, 4 children had positive TPHA antibodies for *T pallidum*. All these prevalence rates are similar or lower compared to available studies conducted in Europe and Italy.^[2,13,16]^ In particular, parasitic intestinal infections were reported in 8% to 38% of cases.^[2,10,13,23,33,34,36]^ In a recent Italian study, parasitological investigation of feces was found to be positive up to 42.7% children.^[16]^ All these studies have described *G lamblia*, *Hymenolepis nana*, *Entamoeba hystolitica*, and *Strongyloides stercoralis* as the most frequent parasitoses, whereas in our cohort *T canis* was the most frequent parasitic pathogen besides *G lamblia*. This could be possibly related to a lack of *T canis* serology in the large majority of the studies.^[2,13,16,32]^ It should be also taken into account that the presence of *T canis* serology (IgG) does not make a distinction possible between current or past infections, and these data only represent an evaluation of seroprevalence. On the other hand, the high prevalence of a positive ova and parasite examination could be related to the high sensitivity reached by the test when performed on 3 stool specimens. In fact, authors have previously confirmed that in high-risk groups of children such as IAC, gastrointestinal symptoms are not predictive of pathogen recovery, and multiple stool specimen evaluations make an increase in pathogen identification possible.^[23]^ Staat and colleagues, in their observational study on 1042 IAC, observed that parasite identification was significantly associated with increasing age, but not with malnutrition and gastrointestinal symptoms. Overall, the yield of 1 stool specimen was 79% with pathogen recovery significantly increasing for 2 (92%) and 3 (100%) specimens, respectively (*P* < 0.0001). Pathogen identification also significantly increased with the evaluation of additional stool specimens for children with and without gastrointestinal symptoms.^[23]^

Differently from our population, in which a prevalence of around 2% was observed for easily treatable cutaneous infections by *M contagiosum* and *T capitis*, a prevalence of cutaneous infections in available studies was reported between 22% and 35%.^[2,10,13,16,23,33,34,36]^

Active TB was diagnosed in 4 children. Nevertheless, latent TB infection (defined with a positive result of TST and QFT or with a positive result either of TST or QFT-G-IT) was diagnosed in 16.4% of the children. This is in line with the 5% to 26% prevalence reported in available literature.^[4,27,30,47–49]^ In the absence of a recognized gold standard, diagnosis of latent TB in children is challenging. It is possible that in the IAC population LTBI may be overestimated in those cases with a positive TST and a negative QFT-G-IT result because of poor TST specificity, due to a cross-reaction with BCG vaccination, booster effect of previous multiple TST tests, and possible nontuberculous mycobacterial infections. In fact, only 3.7% children had concordant TST+/QFT-G-IT+ results; whereas 11.4% had TST+/QFT-G-IT− and 1.3% had TST−/QFT-G-IT+. Similar data were recently reported by Howley and colleagues in 2520 immigrant children screened for latent TB, but with a higher proportion of TST+/QFT-G-IT+: 4.4% were TST+/QFT+, 21.9% were TST+/QFT−, and 1.2% were TST−/QFT+.^[48]^ The authors also reported a significant association with older age and with the presence of TB in at least 1 immigrating family member.^[48]^ In our study, we also observed a higher frequency (13.25% vs 9.15%) of TST+/QFT-G-IT− discordant results in children ≥5 years: this could be related to more prolonged exposure to TB cases in the country of origin (in the large majority of these countries TB is endemic) and to routinely repeated TST in orphanages. In fact, the large majority of children with discordant TST+/QFT-G-IT− results came from eastern-Europe (83/154; 53.9%), where children reside in orphanages and undergo to repeated TST, possibly leading to an increased risk of a positive result. Moreover, we observed in the same population a higher proportion of BCG-vaccinated children versus in those ages <5 (*P* ≤ 0.0001). A significantly higher and independent risk of TST+/QFT-G-IT− with BCG vaccination and age ≥5 years was confirmed in a multivariable analysis (aOR: 2.184 [1.26–3.79]; *P* = 0.006) and (aOR: 1.49 [1.06–2.11]; *P* = 0.02). Association between BCG and positive TST results in children was widely confirmed, in particular in a large meta-analysis, on 117,507 subjects.^[50–52]^

Interferon-gamma-release assays (IGRA) performance in pediatric populations is still under debate and caution is recommended for their use and interpretation in young children.^[53–57]^ For its advantage of not being influenced by BCG, several authors have studied and confirmed the importance of QFT-G-IT especially in BCG-vaccinated children, but on the other hand, children under the age of 2 and nonvaccinated children may display negative results.^[53–57]^ In a systematic review and meta-analysis that we published in 2014, IGRAs showed good promise for improving TB diagnosis only in immunocompetent children >5 years, in a high-income setting. Even in these subjects, however, IGRAs sensitivity was 67% to 86%, indicating that neither test may rule out nor confirm the certainty of diagnosis and, similarly to the TST, interpretation of results may be difficult.^[58]^ It has been described that IGRA performance is influenced by the child's immunologic status, which, in turn, may be impaired by several conditions commonly observed in low-income countries, such as malnutrition, HIV infection, and helminthiasis.^[59–64]^ One limit of our study is that we were not able to consider the subjects’ nutritional status. Moreover, no helminthic infection was described in our population, with the exception of 1 case of *Tenia solium* infection.

Available data on association between parasitic infections and TST and QFT-G-IT results are poor^[34,65]^: Wassie and colleagues screened 245 healthy school children from Addis Abeba, a TB-endemic region, with TST and QFT-G-IT. Although concordance between the tests was generally fair (90%), there was a subset of children who had positive QFT-G-IT with negative TST and a strong association with the presence of parasites in the stool of these patients.^[65]^ Piñeiro-Pérez et al did not found differences in TST results among 1074 infected and noninfected children.^[34]^ In our univariable and multivariable analyses, we did not find an association between parasite infection and positive result of TST or of QFT-G-IT, nor with eosinophilia. However, only 1 case of helminthic infection by *T solium* was found.

In our univariable and multivariable logistic regression analyses, the only factor associated with a positive QFT results was the lack of previous BCG vaccination: this phenomenon could be explained by protection guaranteed by BCG versus TB infection, as previously reported.^[51,52]^ Indeed, as it is demonstrated that BCG vaccination provides a good level of protection against tuberculous meningitis and disseminated forms, and a fair level of protection against pulmonary disease, the possibility for vaccinated children to be TB infected was widely observed and described.^[51,52]^

Concerning vaccinations, the majority of the children underwent specific serologic tests to evaluate protective or nonprotective antibody titer versus tetanus (82.1%), HBV (82.9%), rubella (77.4%), and measles (77.1%), displaying a nonprotective serology in 15.7%, 35.1%, 32.8%, and 25.9% respectively. All these serologies were performed with CLIAs which in previous studies displayed high sensitivity and specificity (≥97.6% and ≥96.6%, respectively, for all the serologies).^[66–70]^

These data are similar to those recently published,^[13,15,17,19]^ confirming that IAC should be tested rapidly for their immunization status on arrival in the adopting country, because they are not protected in a sufficient way against vaccine-preventable diseases. Moreover, preadoptive immunization records are often lacking and scarcely reliable^[13,15,17,19]^: indeed, in our population vaccine documentation was available in only 38.9% to 46.1% of the cases (mostly in European children), with a variable discrepancy between available documentation and test results (ranging from 9% for tetanus and 24.1% for HBV). Some authors reported that in a population of 562 IAC the number of doses recorded was the best predictor of protective antibody titer,^[71]^ but in our study, 52/616 (8.4%) children with ≥3 doses of tetanus vaccine had a nonprotective antibody titer.

Considering the costs related to all the performed serologies, and the cost of revaccinating, the recommendation of empirically revaccinating all internationally adopted children could be reasonable. Clearly, a cost analysis was not one of the purposes of our study; however, our results could encourage further studies focusing on this specific topic that could be useful to optimize present screening strategies.

Our cross-sectional study had some limitations. Dividing the population into subgroups for analyses and comparisons led to limited subsets of patients. Furthermore, during the 7-year study period some investigations included in the screening protocol changed, and some tests were not performed in the whole population: in fact, the large majority of the children referred to our Center and included in the study were evaluated as per protocol, but for some variables we had some missing information possibly due to the physicians’ incomplete adherence to the screening protocol or to an incomplete collection of data in medical records. Indeed, it must be considered that our Center was able to visit only a part of all the adopted children arriving in our region, as some parents (despite the recommendation to see a doctor for all adopted children, regardless of their country of origin) may have consulted their family physician or no one at all.

## 5 Conclusions

Our data underline the importance of a rapid, careful, and complete screening in IAC. Clinical problems have been observed frequently, ranging from congenital malformations, complex infectious diseases, as TB infection (16.7%) and parasitosis (23.1%), to nonsevere and easily treatable infections, as *M contagiosum* and fungal skin infections. Notably, 39.14% children had at least 1 infectious disease, with a total of 743 infectious diseases diagnoses in 631 children. It is important to consider the vaccine documentation and screening of the IAC in order to update immunizations. Moreover, according to our results, the diagnosis of latent TB infection is particularly difficult, due to the high rate of discordant TST/QFT-G results. QFT-G-IT results may be more reliable in children over the age of 5, suggesting the utility in this subset of children of a “wait and see” approach, monitoring the child with QFT-G-IT, to avoid overtreatment.
